# Detection of trypanosomes in small ruminants and pigs in western Kenya: important reservoirs in the epidemiology of sleeping sickness?

**DOI:** 10.1186/1475-9292-4-5

**Published:** 2005-07-14

**Authors:** Musa O Ng'ayo, Zablon K Njiru, Eucharia U Kenya, Geoffrey M Muluvi, Ellie O Osir, Daniel K Masiga

**Affiliations:** 1 Department of Biochemistry and Biotechnology, Kenyatta University, P.O. Box 43844, 00100 – Nairobi, Kenya; 2 Molecular Biology and Biochemistry Department, International Centre of Insect Physiology and Ecology, Duduville, Kasarani, P.O. Box 30772, 00100 – Nairobi, Kenya; 3 Trypanosomiasis Research Centre, Kenya Agricultural Research Institute, P.O. Box 362, Kikuyu, Kenya; 4 Western Australia Biomedical Research Institute, Division of Health Sciences, School of Veterinary and Biomedical Sciences, Murdoch University, Murdoch, WA 6150, Australia

**Keywords:** *Trypanosoma brucei rhodesiense*, Human African Trypanosomosis, sleeping sickness, epidemiology, Serum Resistance Associated (SRA) gene, small ruminants, pigs, reservoirs.

## Abstract

**Background:**

Trypanosomosis is a major impediment to livestock farming in sub-Saharan Africa and limits the full potential of agricultural development in the 36 countries where it is endemic. In man, sleeping sickness is fatal if untreated and causes severe morbidity. This study was undertaken in western Kenya, an area that is endemic for both human and livestock trypanosomosis. While trypanosomosis in livestock is present at high levels of endemicity, sleeping sickness occurs at low levels over long periods, interspersed with epidemics, underscoring the complexity of the disease epidemiology. In this study, we sought to investigate the prevalence of trypanosomes in small ruminants and pigs, and the potential of these livestock as reservoirs of potentially human-infective trypanosomes. The study was undertaken in 5 villages, to address two key questions: i) are small ruminants and pigs important in the transmission dynamics of trypanosomosis? and ii), do they harbour potentially human infective trypanosomes? Answers to these questions are important in developing strategies for the control of both livestock and human trypanosomosis.

**Results:**

Eighty-six animals, representing 21.3% of the 402 sampled in the 5 villages, were detected as positive by PCR using a panel of primers that identify trypanosomes to the level of the species and sub-species. These were categorised as 23 (5.7%) infections of *T. vivax*, 22 (5.5%) of *T. simiae*, 21 (5.2%) of the *T. congolense *clade and 20 (5.0%) of *T. brucei *ssp. The sheep was more susceptible to trypanosome infection as compared to goats and pigs. The 20 *T. brucei *positive samples were evaluated by PCR for the presence of the Serum Resistance Associated (SRA) gene, which has been linked to human infectivity in *T. b. rhodesiense*. Three samples (one pig, one sheep and one goat) were found to have the SRA gene. These results suggest that sheep, goats and pigs, which are kept alongside cattle, may harbour human-infective trypanosomes.

**Conclusion:**

We conclude that all livestock kept in this *T. b. rhodesiense *endemic area acquire natural infections of trypanosomes, and are therefore important in the transmission cycle. Sheep, goats and pigs harbour trypanosomes that are potentially infective to man. Hence, the control of trypanosomosis in these livestock is essential to the success of any strategy to control the disease in man and livestock.

## Background

African trypanosomosis has profoundly affected settlement and economic development in much of the African continent, especially south of the Sahara desert where it is transmitted mainly by tsetse flies. Overlaying a map of the distribution of livestock and that of tsetse infestation highlights the extent to which tsetse and trypanosomosis impede livestock development [1]. Additionally, sleeping sickness or Human African Trypanosomosis (HAT) is responsible for considerable morbidity and mortality in many countries of sub-Saharan Africa. Sleeping sickness is caused by two protozoan parasites of genus *Trypanosoma*: *Trypanosoma brucei rhodesiense *and *T. b. gambiense*. The latter is characterized by a chronic disease in man, often lasting several years, and is distributed mainly in western and central Africa. *T. b. rhodesiense *is more acute, with clinical signs apparent within weeks of infection and is confined to eastern and southern Africa. For a long time, wildlife were considered the most important, perhaps the only reservoir for both sleeping sickness and trypanosomosis in livestock. However, this changed when it was demonstrated that cattle were reservoirs of *T. b. rhodesiense *in Kenya [2]. With respect to *T. b. gambiense *on the other hand, it has been shown that pigs are reservoirs [3], although a direct man-fly-man transmission cycle is considered the more important [4]. The acute nature of sleeping sickness due to *T. b. rhodesiense *appears to preclude this, making the presence of a non-human reservoir obligatory in maintaining an endemic focus. Recent cases from areas where wildlife are protected, such as Serengeti National Park in Tanzania, indicate that wildlife continue to play a role in transmission of sleeping sickness [5].

Unlike cattle, sheep and goats are kept in a very broad range of agro-ecological zones [1], where they contribute considerably to the rural economies as a source of meat, milk, manure and readily disposable income [6]. Early reports suggested that trypanosomosis was not an important disease in small ruminants [7]. However, more recent studies have clearly demonstrated that sheep and goats acquire natural infections [8-12], and suffer economic loss [13,14]. Trypanosomes have also been shown to infect pigs, with reports of natural infections in different regions of Africa [9,11,15,16].

This study was undertaken in western Kenya, an area that is endemic for Human African Trypanosomosis (HAT) or sleeping sickness, and several species of trypanosomes of veterinary importance. We sought to investigate the role of small ruminants and pigs in the transmission dynamics of trypanosomosis. In this area, pigs are increasingly kept as free-range livestock, presumably thriving as a result of the varied nature of their diet. Our efforts to identify *T. b. rhodesiense *have benefited from evidence that the Serum Resistance Associated gene (SRA) is ubiquitous among all *T. b. rhodesiense *isolates tested so far that have also been characterized by other biochemical criteria [17-19].

## Results

### Detection of trypanosomes

The buffy coat and haematocrit centrifuge technique (HCT) [20] examination of blood from the 402 animals detected 5 trypanosome infections as shown in Table 1. The infections detected by microscopy were one *T. congolense*, one *T. vivax *and three *T. brucei*; no mixed infections were detected using microscopy alone. All 402 animals sampled were subjected to PCR analysis for detection of trypanosomes, based on primers specific for different species and subspecies (Table 2). Eighty-six (18.9%) animals out of the 402 were found infected by PCR. Thirty-one (27.7%) out of 112 animals sampled from Obuchun were infected by various species of trypanosomes. The infection rate was 28.05% (23/82) in Amoni, 22.1% (15/68) in Ongariama, 19.2% (10/52) in Amase and 7.9% (7/88) in Rukada. Twenty-four of the 95 sheep sampled (25.3%) were infected, while 52/255 goats (20.0%) and 10/52 pigs (19.2%) were infected. There was no significant difference in infection between animals (*p *> 0.05).

**Table 1:** Identification of trypanosome species using PCR.

**Amoni**								
Goats	42	2	3	1	0	2	8	**19.04**
Sheep	37	0	6	5	0	4	15	**40.54**
Pigs	3	0	0	0	0	0	0	**0**

**Amase**								
Goats	35	0	0	0	4	1	5	**14.29**
Sheep	8	0	0	0	0	1	1	**12.5**
Pigs	9	1	0	0	1	2	4	**44.44**

**Ongariama**								
Goats	55	0	0	7	5	1	13	**23.64**
Sheep	7	0	0	0	0	0	0	**0**
Pigs	6	0	0	1	1	0	2	**33.33**

**Obuchun**								
Goats	86	4	0	8	8	3	23	**26.74**
Sheep	13	0	1	0	1	2	4	**30.77**
Pigs	13	0	0	0	3	1	4	**30.77**

**Rukada**								
Goats	37	1	1	0	0	1	3	**8.11**
Sheep	30	1	1	0	0	2	4	**13.33**
Pigs	21	0	0	0	0	0	0	**0**

**TOTAL**	**402**	**9**	**12**	**22**	**23**	**20**	**86**	**21.39%**

**Table 2:** Sequences of primers used for PCR and expected product size

**Trypanosome species**	**Primer sequence 5'-3'**	**Size (bp)**	**Reference**
*T. Congolense *savannah	For: CGAGAACGGGCACTTTGCGA	316	[28]
	Rev: GGACAAACAAATCCCGCACA		
*T. congolense *Kilifi	For: GTGACCAAATTTGAAGTGAT	294	[28]
	Rev: ACTCAAAATCGTGCACCTCG		
*T. vivax*	For: CCCGGCAGGTTGGCCGCCATC	399	[29]
	Rev: TCGCTACCACAGTCGCAATCGCAATCGTCGTCTCAAGG		
*T. simiae*	For: CCGGTCAAAAACGCATT	437	[28]
	Rev: AGTCGCCCGGAGTCGAT		
*T. brucei*	For: GAATATTAAACAATGCGCAG	164	[28]
	Rev: CCATTTATTAGCTTTGTTGC		
*SRA – T. b. rhodesiense*	SRA-A: 5'GACAACAAGTACCTTGGCGC	460	[18]
	SRA-E: 5'TACTGTTGTTGTACCGCCGC)		

### Prevalence of trypanosome species

The prevalence of *Trypanosoma vivax *as detected by PCR was 23 (5.7%), *T. simiae *22 (5.5%), *T. congolense *savannah 9 (2.2%), *T. congolense *Kilifi 12 (3.0%) and *T. brucei *ssp 20 (5.0%). A number of mixed infections were reported in this study; *T. congolense *Savannah and *T. brucei *were encountered twice, and there was one mixed infection of *T. congolen*se savannah and *T. simiae*. Sheep carried significantly more infections of *T. congolense *Kilifi than goats (χ^2 ^= 9.8, df = 1, *p *< 0.05) and similarly with *T. brucei *ssp (χ^2 ^= 6.6, df = 1, *p *< 0.05). There were more *T. vivax *infections in goats and pigs than in sheep respectively, and this difference was significant (*p *< 0.05).

### Presence of Serum Resistance Associated (SRA) Gene

**Figure 1:**
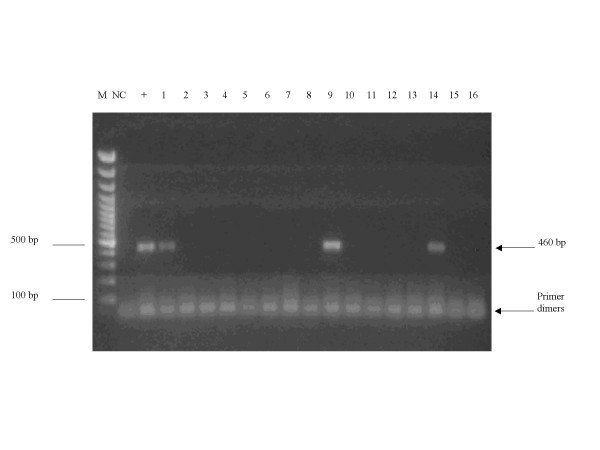
**Map of study area showing sampling locations. **Sampling was undertaken in 5 villages (4 in Teso and 1 in Busia Districts), whose GPS locations are shown in this map.

Twenty samples in which T. brucei spp. was detected by PCR were further analysed for the presence of the SRA. This analysis resulted in 3 positive samples for the SRA gene (from a goat, pig and sheep) (Figure 1), demonstrating the presence of this gene associated with human infectivity in trypanosomes infecting livestock other than cattle.

## Discussion

### Trypanosomes in small ruminants

Few records exist on the effect of trypanosomosis in small ruminants in East Africa. However, the work of Griffin and Allonby [8,13], who used microscopic examination of blood smears to demonstrate that the disease was an important natural infection, is a notable exception. These authors also reported that small ruminants survived better than cattle under medium tsetse challenge. More recent studies undertaken in an area of high tsetse fly challenge have shown that small ruminants succumb to trypanosomosis and that heavy economic loss is occasioned [12,14]. Although the microscopic techniques used in these studies have limitations of sensitivity [20], they provided an important basis for investigations into the importance of trypanosomosis in small ruminants. In the present study, using PCR raised the number of infections detected from 5 to 86, considerably improving our understanding of the prevalence of trypanosomosis in small ruminants and pigs.

A greater percentage of sheep were infected compared to goats and pigs, findings that are consistent with other studies that have shown sheep to be more frequently infected than goats under natural conditions [9,11,12], [21-24]. All these findings, drawn from experiments on different breeds suggest that goats are more refractory to trypanosome infections than sheep. The presence of many small ruminants with *T. simiae *(5 sheep and 16 goats) shows that this trypanosome has the potential to impact negatively on the growing importance of pig farming in the study area. Our data also show that sheep were more frequently infected with *T. congolense *Kilifi and *T. brucei*, while pigs and goats were more frequently infected with *T. vivax*.

All the 5 villages, except Ongariama, had infections of *T. congolense*, arguably the most important pathogens of cattle in Africa. Also, with the exception of Rukada and Amoni, *T. vivax *infections were identified. These two species constitute the most important trypanosomes of livestock in Africa. Considering the greater trypanotolerance of small ruminants, they may be acting as reservoirs of these pathogens, therefore limiting the economic impact of the cattle industry.

### Trypanosome infections in pigs

The presence of trypanosomes in pigs has been documented from different parts of Africa [9,11,15,16]. In the present study, out of the 10 pigs infected, 3 carried *T. brucei *ssp., 5 had *T. vivax*, and there was one infection each of *T. congolen*se savannah and *T. simiae*. Of important relevance to sleeping sickness epidemiology, the SRA gene was detected in one pig carrying *T. brucei*. These data show that pigs play a role in the epidemiology of both human and animal trypanosomosis.

### Reservoirs of sleeping sickness

Small ruminants and pigs constitute a key component of livestock kept in the study area. It is now well recognized that these livestock naturally acquire trypanosome infections. On the basis of DNA amplification of the SRA gene, this study shows that sheep, goats and pigs may harbour *T. b. rhodesiense*. It is in this context that investigations were undertaken in these livestock as potential reservoirs of *T. b. rhodesiense*. Reports implicating cattle date back to the infection of human subjects with pathogens isolated from cattle [2]. In the intervening period, descriptions of human infective trypanosomes in cattle have been made on the basis of multilocus DNA fingerprinting [15], and more recently the presence of the SRA gene as a marker [17,18]. The epidemiology of HAT in western Kenya is very complex, and key factors can vary within very short geographical distances. The existence of two species of tsetse flies, *G. pallidipes *and *G. fuscipes fuscipes*, which have different habitats, and feeding preferences, adds to this complexity. *G. fuscipes fuscipes *feeds primarily on the Nile monitor lizard, *Varanus niloticus *[25]. This probably prompted experiments that led to the isolation of *T. brucei *from one monitor lizard [26], but the ability of these cold-blooded reptiles to support trypanosomes, other than Stercorarian forms such as *T. grayi *remains unconfirmed.

The ability to identify *T. b. rhodesiense *in reservoirs and tsetse provides a means for estimating disease risk, even in the absence of current human infections. Our study also identified one pig that was infected with trypanosomes bearing the SRA gene. It is estimated that there are more than 10,000 pigs reared in a free-range management system in the area (Dr. Samuel Maina, personal communication). Their large number, and the evidence that they can act as reservoirs underscores the importance of this study on the health and economic development of rural Africa, even on a small geographical scale.

## Conclusion

To date, no significant efforts are made, either by the relevant government agents, or farmers to control trypanosomosis in small ruminants and pigs. This study contributes to the understanding of the epidemiology of sleeping sickness and livestock trypanosomosis in this area, with implications for the entire lake Victoria basin. Our conclusion is that small ruminants and pigs may constitute an enduring reservoir of trypanosomes that infect both humans and their livestock. Concerted efforts to control trypanosomes in these livestock can contribute significantly to a multi-disciplinary approach to trypanosomosis control in western Kenya, and other areas where the disease is endemic, and have an impact on the well being of the inhabitants of this region.

## Methods

### Study area

**Figure 2:**
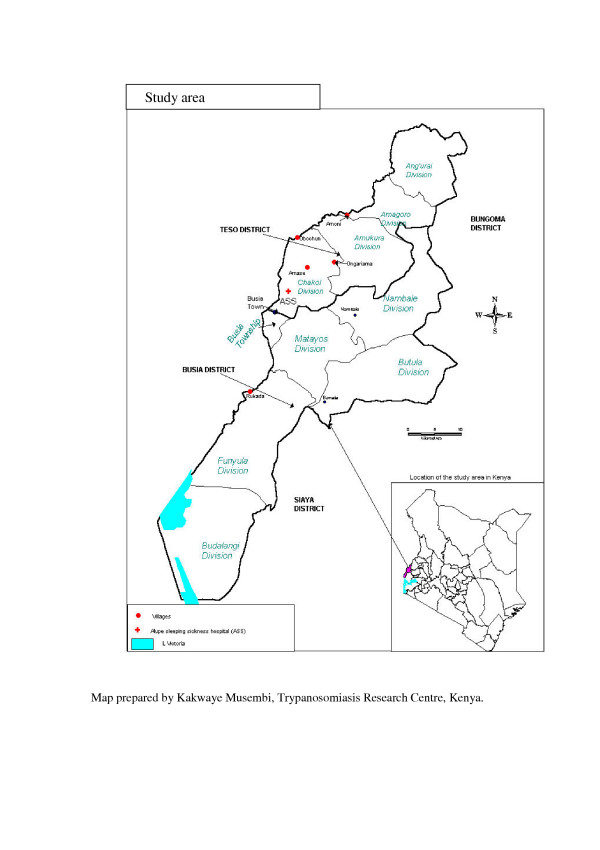
**Detection of SRA gene. **Ethidium Bromide stained 1.5% agarose gel showing PCR identification of samples containing putative *T. b. rhodesiense *using SRA A and E primers. M, 100 bp DNA marker; NC, Negative Control, +, positive control. Lanes 1 (pig), 9 (sheep) and 14 (goat) show the expected fragment of 460 bp, which indicates the detection of the SRA gene in these samples.

The study area included two districts in western Kenya, Busia and Teso. Sampling was carried out in five villages: Rukada in Busia district; Amoni, Amase, Obuchun and Ongariama in Teso district (Figure 2). These villages were selected on the basis of having reported cases of sleeping sickness within a period of about 10 years prior to the study. Samples were collected in August 2001 over a period of two weeks. The two districts lie between latitude 0° 1'South and 0° 46' North; and longitude 33° 54' West and 34° 26' East (Figure 2). The altitude varies from 1130 m to 1375 m. The mean annual rainfall is 1500 mm divided between two seasons, while annual maximum temperature ranges from 26°C to 30°C and minimum temperatures vary from 14°C to 18°C. Busia District borders Lake Victoria and the entire study area is traversed by two tributaries (rivers Sio and Malaba) and several streams that feed into them. It is a mixed farming area, with a variety of livestock species, mainly cattle, sheep and goats. Over the past two decades, free-range pig rearing has increased steadily. Two species of tsetse flies (Diptera: *Glossinidae*) infest the area. *G. fuscipes fuscipes*, which is a riverine species, associated with rivers/streams and the shores of Lake Victoria, while *G. pallidipes *is more widely distributed and associated with shrubs, thickets and open grassland. The distribution of *G. pallidipes *is discontinuous and occurs in several small foci of infestation (Kenya Trypanosomiasis Research Institute, unpublished). In areas infested with *G. pallidipes*, bushes of *Lantana camara*, a suitable habitat for resting and larviposition are widespread.

### Animal sampling

Blood samples were collected from 402 animals (255 goats, 95 sheep and 52 pigs) that were brought to the sampling site by local farmers. The animals were bled from the ear vein into two capillary tubes. One capillary tube was used to estimate the percentage Packed Cell Volume (%PCV) and microscopic examination for trypanosomes, and also to make an initial diagnosis on the basis of morphology and movement [20,27]. Animals were also bled from the jugular vein into 2 ml vials containing EDTA, and stored in liquid nitrogen for further analysis.

### Template preparation and PCR cycling

For each sample, 500 μl of cryopreserved blood was thawed into a single 1.5 ml microcentrifuge containing 500 μl of Saponin lysis buffer (0.15% w/v Saponin, 0.2% w/v NaCl and 1 mM EDTA) and mixed by vortexing. The mixture was then centrifuged at 11,000 g for 10 min in a microcentrifuge, followed by four washes in the same buffer. The resulting pellet was then resuspended in 100 μl of PCR buffer (50 mM KCl, 1.5 mM MgCl_2 _10 mM Tris-Cl, pH 8.3) and incubated at 95°C for 20 minutes, cooled and stored at -20°C for.

Standard PCR cycling was carried out in 25 μl reaction mixtures containing; 75 mM Tris-HCl, pH 8.8, 20 mM (NH_4_)_2_SO_4_, 0.1 % Tween 20), 200 μM of each of the four deoxynucleoside triphosphates (dNTPs), primers at 1 μM, 1 μl DNA template (except for screening the presence of SRA gene, where 4 μl of template was used), and 1 unit of *Taq *DNA polymerase (Fermentas MBI, Lithuania). The PCR cycling involved an initial denaturation step at 94°C for 3 minutes, followed by 30 cycles of denaturing at 94°C for 30 s, annealing at 60°C for 45 s and extension at 72°C for 30 s, with a final elongation step at 72°C for 5 minutes. Five microlitres of each PCR product was mixed with standard loading dye (Fermentas MBI, Lithuania) and electrophoresed in 1.5% agarose, with ethidium staining (5 μg/ml) and photographed under ultraviolet illumination. A positive control (with reference DNA) and a negative control (without DNA) were included with each set of reactions. PCR primers and their source references are given in Table 2.

### Statistical analysis

Statistical analysis was performed using SPSS v11. The prevalence of trypanosome species and infection between animals were determined using chi-square test and odds ratios and their 95% confidence intervals. The level of significant difference was set at 0.05.

## Competing interests

The author(s) declare that they have no competing interests.

## Authors' contributions

MNO undertook this study as part of his training for the Master of Science degree at Kenyatta University, where he was supervised by EUK and GMM. Field and laboratory activities were jointly supervised by ZKN (at KETRI), DKM and EOO (at ICIPE). All authors read and approved the final manuscript.
